# Supplementary figures and images for: Unravelling induced resistance in strawberry: distinct metabolomic signatures define cultivar-specific resistance to Botrytis cinerea
Source: Front Plant Sci. 2025 Sep 26;16:1675649. doi: 10.3389/fpls.2025.1675649 (PMC12510941; doi:10.3389/fpls.2025.1675649)

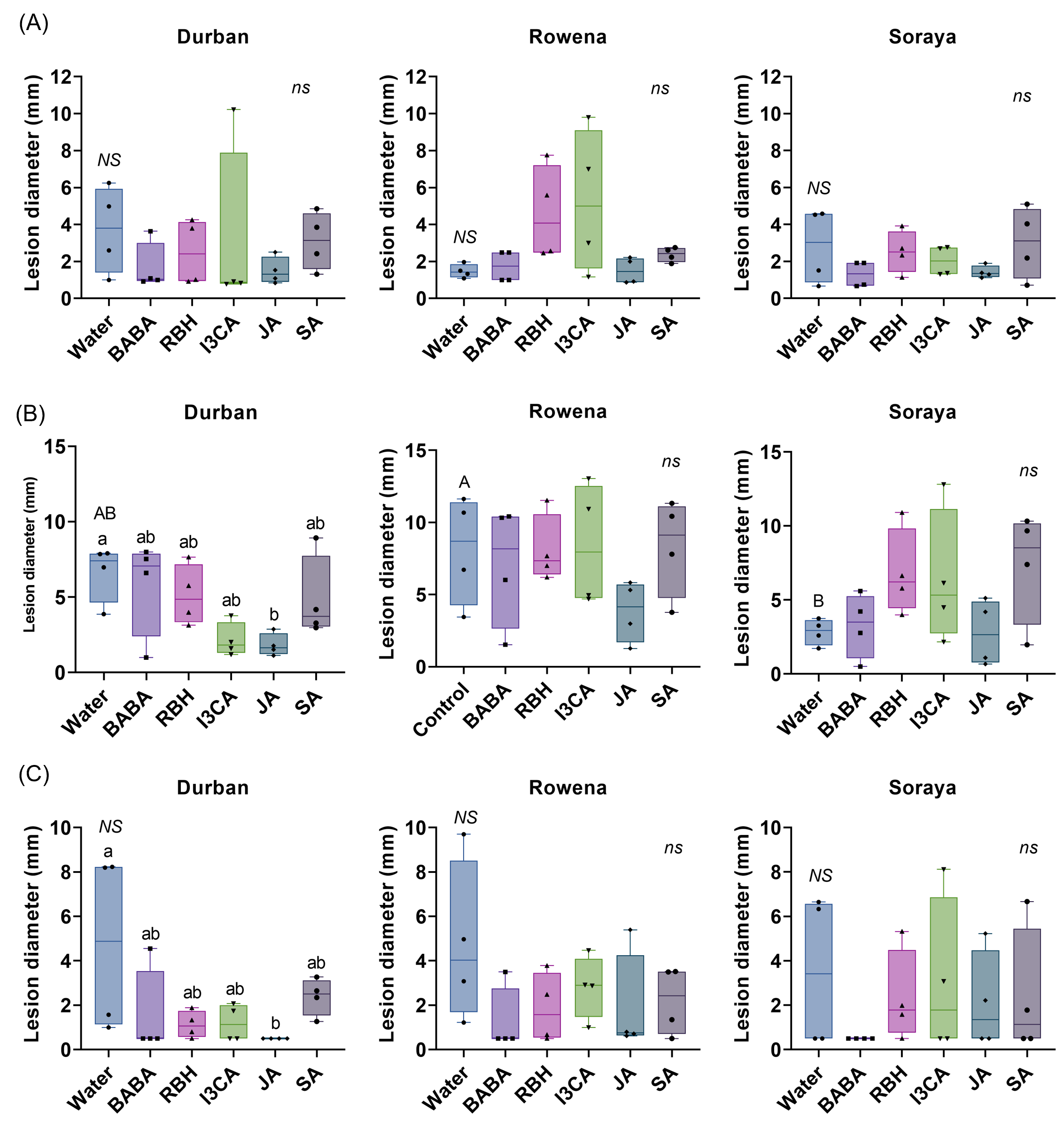

Supplement: Supplementary Figure 1 — Lesion development in strawberry cultivars during early elicitor applications. Lesion diameter in Durban, Rowena, and Soraya after the first (A), second (B), and third (C) elicitor applications at 6, 8, and 10 weeks of plant age, respectively. Inoculations were performed five days after each treatment application. Boxplots represent lesion diameter (mm) with the median line, interquartile range (boxes), and whiskers extending to the minimum and maximum values. Each point represents biological replicates (individual plants) (n = 8–12 per treatment). Capital letters indicate statistical differences between the Water treatments of each variety. Lowercase letters indicate statistically significant differences between elicitors within each variety (One-way ANOVA followed by Tukey´s post hoc test or Welch’s ANOVA followed by Dunnett’s T3; p < 0.05; n = 8–12). [file Image1.tif]

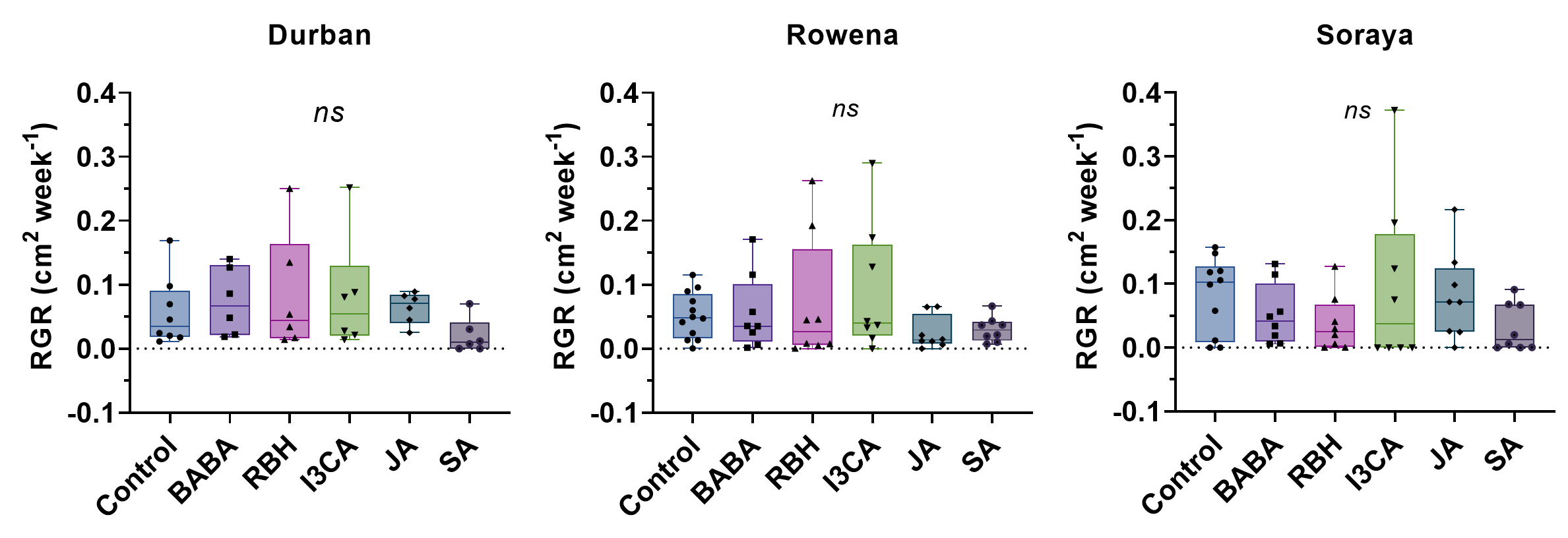

Supplement: Supplementary Figure 2 — Assessment of growth following elicitor treatments. Relative Growth Rate (RGR) was calculated based on leaf area expansion between weeks 11 and 14 using ImageJ software. Boxplots represent RGR (cm2 week-1) with the median line, interquartile range (boxes), and whiskers extending to the minimum and maximum values. Each point represents biological replicates (individual plants). ns indicates not significant differences (One-way ANOVA; p < 0.05; n = 8–12). [file Image2.tif]

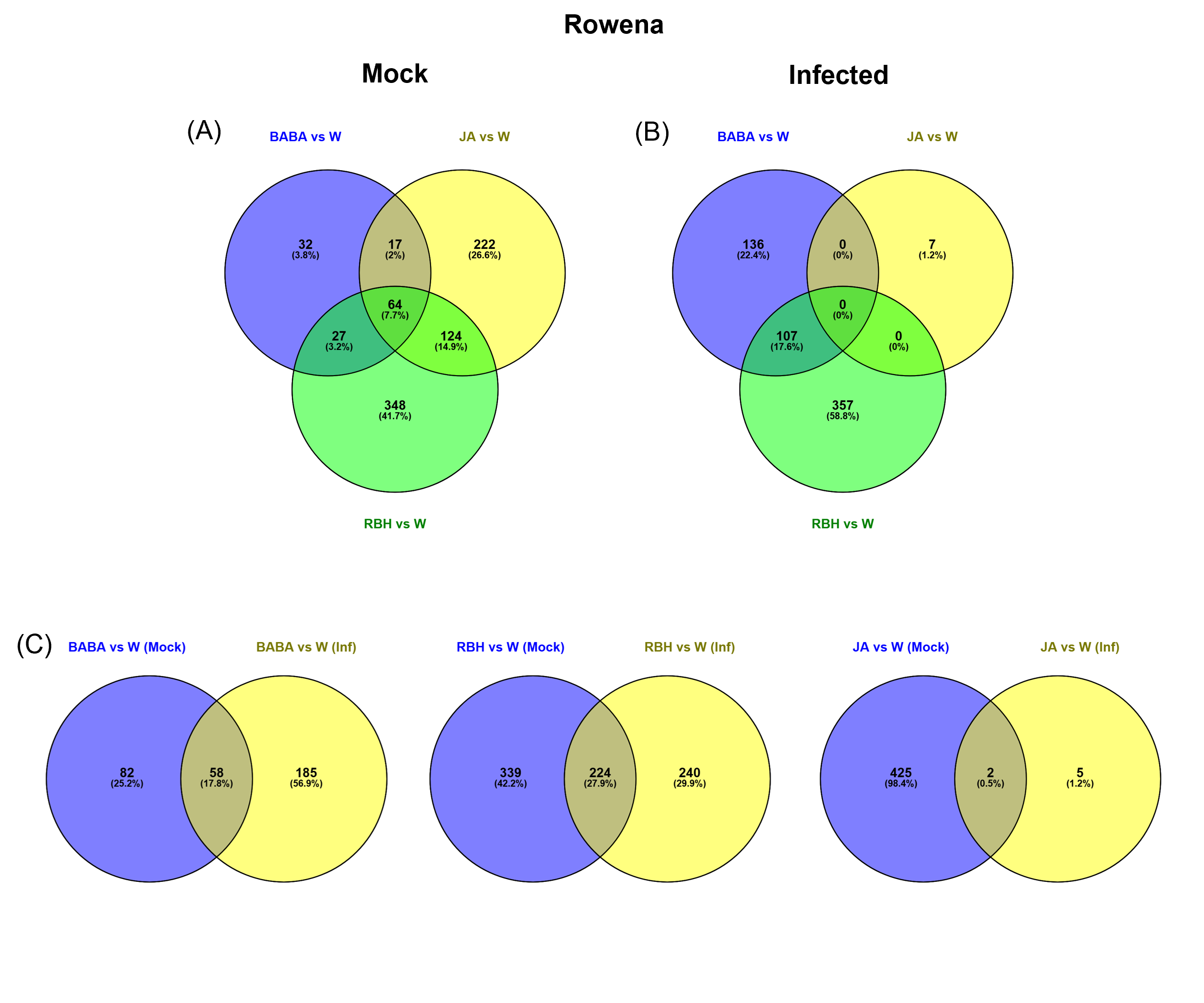

Supplement: Supplementary Figure 3 — Metabolite reprogramming in Rowena in response to elicitors. (A) Venn diagram showing metabolites induced under mock condition by BABA, RBH, and JA in Rowena. (B) Venn diagram showing metabolites induced under B. cinerea infected condition. (C) Filtering of mock- (blue circles) and infection (yellow circles)-specific metabolites for each treatment. The diagram separates direct (mock-only) and priming (infection-only) responses, prior to exclusive grouping in Figure 4 . [file Image3.tif]

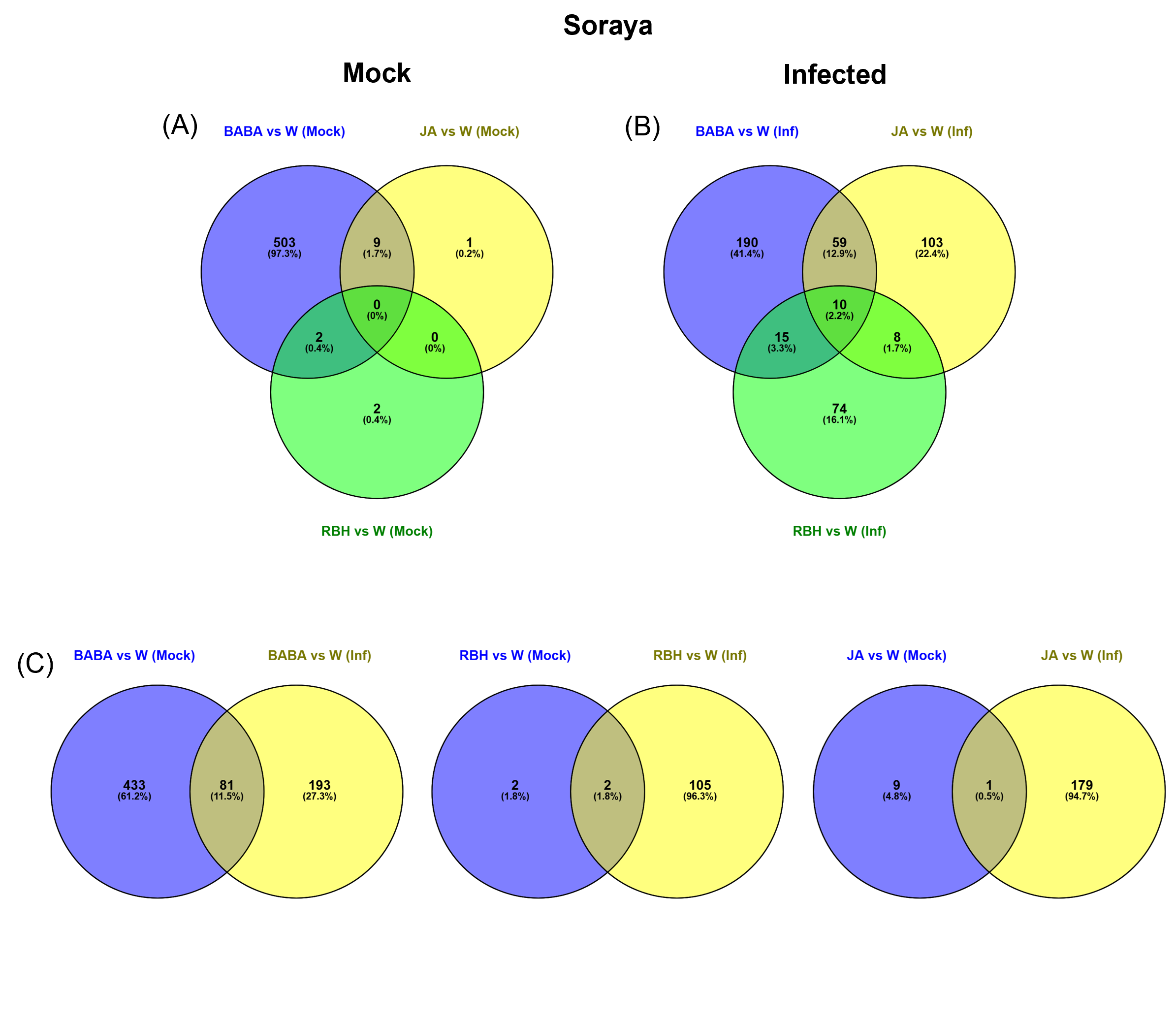

Supplement: Supplementary Figure 4 — Metabolite reprogramming in Soraya in response to elicitors. (A) Venn diagram showing metabolites induced under mock condition by BABA, RBH, and JA in Soraya. (B) Venn diagram showing metabolites induced under B. cinerea infected condition. (C) Filtering of mock- (blue circles) and infection (yellow circles)-specific metabolites for each treatment. The diagram separates direct (mock-only) and priming (infection-only) responses, prior to exclusive grouping in Figure 5 . [file Image4.tif]
