# Supplementary material for: Unravelling induced resistance in strawberry: distinct metabolomic signatures define cultivar-specific resistance to Botrytis cinerea
Source: Front Plant Sci. 2025 Sep 26;16:1675649. doi: 10.3389/fpls.2025.1675649 (PMC12510941; doi:10.3389/fpls.2025.1675649)
Supplement: Supplementary file 5 [file DataSheet1.docx]

**Supplementary Table 1.** Mean ± SEM values of the number of flowers per cultivar under each treatment. Flowers were counted after the third elicitor application (week 11 of the experiment) and the fourth elicitor application (week 13 of the experiment). Different letters indicate statistically significant differences between the treatments within each cultivar. (One‐way ANOVA followed by Tukey´s post hoc test ; p < 0.05; n = 8–12).

| Time (week) | Cultivar | Control | BABA | RBH | I3CA | JA | SA |
| --- | --- | --- | --- | --- | --- | --- | --- |
|  | Durban | 4.12±2.03 | 4.16±1.60 | 2.00±1.00 | 3.20±0.83 | 3.66±1.36 | 3.80±2.58 |
| 11 | Rowena | 1.3±0.50 | 2.25±0.95 | 2.00±1.15 | 2.42±0.78 | 1.50±0.57 | 2.20±0.83 |
|  | Soraya | 2.40±1.42 | 3.33±1.15 | 3.00±1.30 | 2.66±1.36 | 3.66±1.86 | 1.80±0.44 |
|  | Durban | 3.25±1.58 | 4.00±1.26 | 2.60±0.54 | 3.16±1.72 | 3.33±0.51 | 3.40±2.07 |
| 13 | Rowena | 1.14±0.37**^a^** | 1.83±0.75**^ab^** | 2.25±0.95**^ab^** | 3.25±1.25**^b^** | 2.00±1.00**^ab^** | 1.60±0.54**^a^** |
|  | Soraya | 1.91±1.16 | 3.33±1.52 | 2.57±1.13 | 2.33±1.21 | 3.50±1.87 | 1.40±0.54 |

**Supplementary Table 2.** Mean ± SEM values of the number of fruit per cultivar under each treatment. Fruit were counted after the fourth elicitor application (week 18 of the experiment). No statistical significant differences were found between the treatments within each cultivar (One‐way ANOVA followed by Tukey´s post hoc test ; p < 0.05; n = 8–12).

| Cultivar | Control | BABA | RBH | I3CA | JA | SA |
| --- | --- | --- | --- | --- | --- | --- |
| Durban | 2.33±1.52 | 0 | 0 | 1.00±0.00 | 1.33±0.57 | 1.34±0.58 |
| Rowena | 1.20±0.44 | 2.00±0.00 | 0 | 2.00±1.41 | 1.00±0.00 | 1.75±1.50 |
| Soraya | 2.25±1.25 | 0 | 0 | 3.50±0.70 | 2.00±1.00 | 1.25±0.50 |
